# Extracorporeal cardiopulmonary resuscitation: a comparison of two experimental approaches and systematic review of experimental models

**DOI:** 10.1186/s40635-024-00664-1

**Published:** 2024-09-13

**Authors:** Anthony Moreau, Fuhong Su, Filippo Annoni, Fabio Silvio Taccone

**Affiliations:** 1 Department of Intensive Care, Erasme hospital, Hopital Universitaire de Bruxelles (HUB), Route de Lennik, 808, 1070 Brussels, Belgium; 2 Laboratoire Expérimental des Soins Intensifs, Université libre de Bruxelles (ULB), Brussels, Belgium

**Keywords:** Extracorporeal cardiopulmonary resuscitation, Cardiac arrest, Brain, Neuromonitoring, Pig

## Abstract

**Background:**

In patients requiring extracorporeal cardiopulmonary resuscitation (ECPR), there is a need for studies to assess the potential benefits of therapeutic interventions to improve survival and reduce hypoxic-ischemic brain injuries. However, conducting human studies may be challenging. This study aimed to describe two experimental models developed in our laboratory and to conduct a systematic review of existing animal models of ECPR reported in the literature.

**Results:**

In our experiments, pigs were subjected to 12 min (model 1) or 5 min (model 2) of untreated ventricular fibrillation, followed by 18 min (model 1) or 25 min (model 2) of conventional cardiopulmonary resuscitation. Results showed severe distributive shock, decreased brain oxygen pressure and increased intracranial pressure, with model 1 displaying more pronounced brain perfusion impairment. A systematic review of 52 studies, mostly conducted on pigs, revealed heterogeneity in cardiac arrest induction methods, cardiopulmonary resuscitation strategies, and evaluated outcomes.

**Conclusions:**

This review emphasizes the significant impact of no-flow and low-flow durations on brain injury severity following ECPR. However, the diversity in experimental models hinders direct comparisons, urging the standardization of ECPR models to enhance consistency and comparability across studies.

**Supplementary Information:**

The online version contains supplementary material available at 10.1186/s40635-024-00664-1.

## Background

Sudden cardiac arrest (CA) is a significant contributor to global mortality and neurologic disability [1–3]. Over recent years, substantial efforts have been made to enhance the quality of cardiopulmonary resuscitation (CPR), including the implementation of early defibrillation [4]. However, even with immediate CPR in witnessed arrests, conventional CPR may remain ineffective for some patients, who might instead benefit from extracorporeal cardiopulmonary resuscitation (ECPR) [5]. By minimizing hypoxic-ischemic brain injury (HIBI) and providing more time to diagnose and treat the potential underlying cause of CA, ECPR may improve survival and neurological outcomes; yet, the effectiveness of such intervention is still debated, with relatively limited data available and many unanswered questions regarding its optimal management [6–8].

Post-cardiac arrest syndrome (PCAS) includes post-cardiac arrest HIBI, post-cardiac arrest myocardial dysfunction, systemic ischemia/reperfusion response with potential capillary leakage, and the ongoing precipitating pathology [9]. Post-cardiac arrest shock is observed in 68% of out-of-hospital cardiac arrest (OHCA) patients; overall mortality rate is around 70%, with most deaths attributed to neurological injury [10]. The frequency and severity of PCAS are largely influenced by the delay in initial treatment, the effectiveness of resuscitation, and the time between collapse and the return of spontaneous circulation (ROSC) [11].

The pathophysiology of HIBI is intricate and is characterized as a "two-hit" model (e.g., primary and secondary injury) [12–18]. Briefly, in the first phase, the cessation of cerebral oxygen delivery results in a disruption of adenosine triphosphate (ATP) production [15], leading to dysfunction of energy-dependent ion channels and anaerobic metabolism. In the second phase, during resuscitation or immediately after ROSC, secondary brain injury is induced by an imbalance between oxygen delivery and consumption, potentially exacerbated by reperfusion, microcirculatory dysfunction, anemia, hypoxia/hyperoxia, hyperthermia, hypocapnia/hypercapnia, and hypotension [12]. Patients requiring ECPR due to refractory cardiac arrest exhibit more severe ischemic and reperfusion cerebral injuries due to prolonged resuscitation and enhanced reperfusion.

In ECPR patients, there is a critical need for comprehensive studies to assess the potential benefits of therapeutic interventions in order to improve survival and reduce HIBI. These interventions could range from physiological—pharmacological treatments to advanced medical devices. However, conducting human studies may be challenging. As the matter of fact, there is a need for a large sample size within a relatively short timeframe to ensure that the results are statistically significant and applicable to the broader patient population. Additionally, there is a necessity to implement multimodal neuromonitoring for each patient with a risk of neurological complications. Establishing such comprehensive monitoring systems for every patient in a clinical study is complex and costly. Therefore, the use of animal experimental models is essential. Animal models provide a controlled environment where researchers can investigate the effects of different therapeutic interventions. Furthermore, these animal studies help to identify the most promising therapies and optimize their application, ultimately leading to improve neurological outcomes and higher survival rates for patients undergoing ECPR.

In this review, we reported the results of two different animal models of experimental refractory CA models implementing ECPR developed in our laboratory. Also, we performed a review of all existing ECPR large animal models published in the literature.

## Methods

### Experimental setting

The Institutional Review Board for Animal Care of the Université libre de Bruxelles (ULB, Belgium) approved all experimental procedures (Ethical Committee approval number: 731 N), which were also in compliance with ARRIVE (Animal Research: Reporting in Vivo Experiments) guidelines. Care and handling of the animals were in accord with National Institutes of Health guidelines (Institute of Laboratory Animal Resources). For all experiments, swine weighing 45–55 kg (*Sus Scrofa Domesticus*) were used.

### Animal preparation

Before the experiment, the animal fasted for 12 h with free access to water. After sedation in the cage with an intramuscular injection of midazolam (1 mg/kg) and ketamine (10 mg/kg) in the neck, the animal was placed on the operating table in a supine position. After establishing electrocardiogram monitoring and cannulating a marginal ear vein, rapid induction for orotracheal intubation using propofol (1.5 mg/kg), atropine (0.5 mg), morphine (3 µg/kg), and rocuronium (1.2 mg/kg) was initiated. Mechanical ventilation was started in controlled volume mode with standardized settings: tidal volume of 8 mL/kg, positive end-expiratory pressure of 5 cmH2O, fraction of inspired oxygen (FiO2) of 100%, and inspiratory to expiratory ratio of 1:2. Respiratory rate and FiO2 were then adjusted to provide a partial pressure of carbon dioxide (PaCO2) and a partial pressure of oxygen (PaO2) between 35 and 45 mmHg and 90 and 120 mmHg, respectively. A continuous venous infusion of rocuronium (1.5 mg/kg*h), morphine (0.2 mg/kg*h), and balanced crystalloids (300–500 ml/h, adapted to obtain a pulse pressure variation < 13%) were started, and a continuous inhaled sevoflurane was administered to achieve an expiratory percentage between 1.8 and 2.3%. With the help of ultrasound echography, a three-lumen central venous catheter was placed in the right external jugular, an arterial catheter in the right radial artery, and an introducer for a pacing wire and a pulmonary artery catheter in the left external jugular. After an incision in the lower abdomen, a Foley catheter was surgically inserted into the bladder. Before the beginning of the instrumentation, 1 g of amoxicillin-clavulanate were administered to the animal.

### Neurosurgical procedure

A complete description of the implementation of multimodal neuromonitoring (MNM) developed in our experimental laboratory was published previously [19]. Briefly, after turning the animal in a prone position, a reverse mirrored F incision was executed on both sides, approximately 0.5 cm from the midline. Using a drill, one hole was made in the skull and one catheter allowing the simultaneous collection of cerebral temperature (CT), intracranial pressure (ICP), and brain oxygen pressure (PbtO2; Neurovent PTO2, Raumedic AG, Germany) was introduced.

### Cannulation procedure and ECMO preparation

After turning the animal back in a supine position, one arterial (17Fr) and one venous (25Fr) cannula (Medtronic, Minneapolis, MN, USA) were placed under echographic control in the femoral artery and vein, respectively. Immediately after cannulation, a bolus of unfractionated heparin (100 UI/kg) was administered, followed by continuous venous infusion (50 UI/kg*h). The cannulas remained clamped till the start of the ECPR and were flushed repeatedly with 50 ml of balanced crystalloids to avoid clot formation. At ECPR initiation, cannulas were then connected to the extra-corporeal membrane oxygenation (ECMO) device. The ECMO circuit included a tubing set (EUROSETS, Medolla, MO, Italy), an oxygenator (EUROSETS, Medolla, MO, Italy), a centrifugal pump (affinity pump, Medtronic, Minneapolis, MN, USA), and a console (Transonic, Ithaca, NY, USA). The ECMO circuit was previously primed with balanced crystalloids.

### Cardiac arrest procedure

**Fig. 1:**
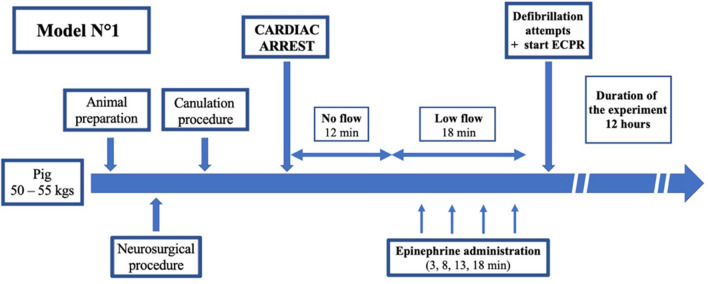
Protocol description of the Model 1. After animal preparation including neurosurgical and canulation procedures, cardiac arrest was induced. Thirty minutes after CA (12 min of no-flow and 18 min of low-flow), defibrillation attempts were performed and ECMO was initiated. The whole experiment lasted 12 h

**Fig. 2:**
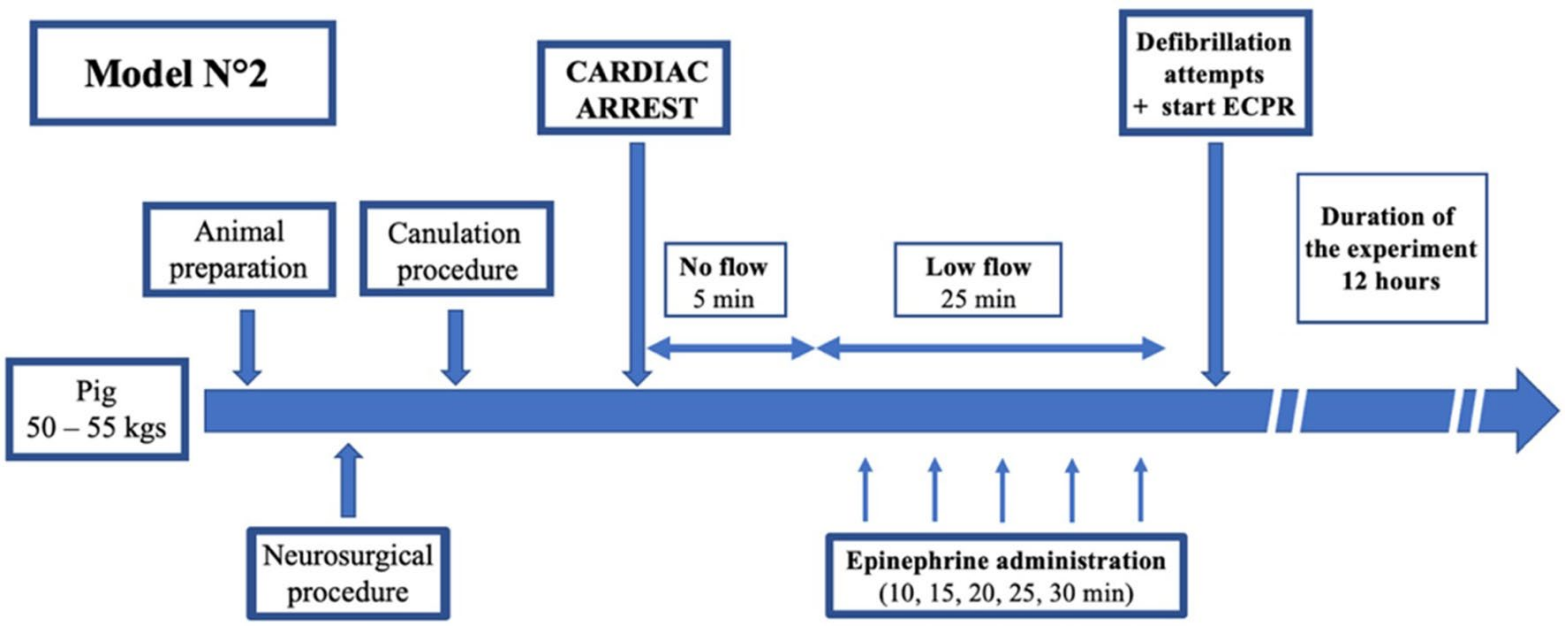
Protocol description of the Model 2. After animal preparation including neurosurgical and canulation procedures, cardiac arrest was induced. Thirty minutes after CA (5 min of no-flow and 25 min of low-flow), defibrillation attempts were performed and ECMO was initiated. The whole experiment lasted 12 h

After a period of stabilization, VF was induced by a catheter introduced into the right ventricle through the left external jugular and connected to a 9 V battery. Animals were therefore allocated to two different ECPR approaches. In the first approach, following CA, the animal was left untreated for a 12-min period (no-flow) and chest compressions were administered for 18 min (low-flow) using a mechanical chest compression device (Lucas III, Jolife AB/Stryker Lund, Sweden) at a rate of 100 compressions per minute. Epinephrine was administered intravenously at intervals of 3, 8, 13, and 18 min during mechanical compressions, each time at a dose of 30 µg/kg, followed by a flush of 10 ml of crystalloid solution. Thirty minutes after the induction of CA, mechanical compressions were halted and ECMO was initiated (initial settings: blood flow 50 ml/kg*min, sweep gas flow 3 L/min and FO2 100%) and defibrillation attempts (4 J/kg biphasic electric shock) were performed, as necessary. In the second approach, no-flow and low-flow periods were 5 and 25 min, respectively (Figs. 1 and 2); epinephrine was administered intravenously at intervals of 5, 10, 15, 20, and 25 min during mechanical compressions. ECMO was initiated at 30 min after induction of CA, using the same settings as above.

ROSC was defined as the establishment of an organized cardiac rhythm with a mean arterial pressure (MAP) exceeding 60 mmHg for a minimum of 10 min. Sweep gas flow was therefore adjusted to maintain a PaCO2 of 35–45 mmHg. A continuous infusion of norepinephrine was given to maintain a MAP between 65 and 70 mmHg. If supraventricular arrhythmia was observed, intravenous amiodarone (300 mg) was administered. Targeted temperature management was facilitated using a heat exchanger connected to the ECMO circuit, aiming 37 °C. Neurological, hemodynamic and respiratory parameters were recorded hourly post-ROSC. Twelve hours post-ROSC, the animal was euthanized via an intracardiac potassium injection.

### Systematic review

A comprehensive, computer-based literature search of the Medline database was performed on the PubMed platform from February 1, 2001 to September 1, 2022. The full PubMed search strategy is provided in the Supplementary material (Table S1). Only studies including large animals (i.e., dogs, swine, sheep) implementing ECPR in the setting of CA were eligible, regardless of the use of different interventions and/or treatments. The outcome of the review was to report the use of ECPR in such models, specifically reporting no-flow and low-flow times, timing of initiation of ECPR and measured outcomes on neurological function (including cerebral blood flow—CBF, intracranial pressure—ICP and/or brain tissue oxygenation—PbtO_2_). Only articles written in English were considered eligible.

All the results obtained from the search were compiled in an Excel datasheet and were independently screened according to the PICO and eligibility criteria by two authors (AM and FS). Any discrepancies were resolved through discussion with a third author (FST). The selection process was completed following the PRISMA recommendations [20]. Initially, all duplicates were removed, and a first selection was made by title and abstract. The remaining articles were assessed for eligibility by full-text, and a brief justification was reported for exclusion. The Cohen’s kappa value of agreement between the two authors (AM and FS) was 0.86. As the aim of the review was purely descriptive, no assessment of bias was performed.

### Statistical methods

Statistical analysis was performed using Prism 9 (Version 9.1.2, San Diego, CA, USA). Continuous variables were expressed as mean with standard deviation (SD). For the comparison of the two models, parametric t-test or a mixed-effects model with Greenhouse–Geisser correction were used as appropriate. The effects of time and group, as well as interactions between groups and time, were tested as fixed effects and animals were introduced as random effects. If there were significant differences, the two-stage linear procedure of Benjamini, Krieger, and Yekutieli, with individual variances, was used to compare the means of these variables for the groups at each time point.

## Results

### Comparison of two different ECPR models

**Fig. 3:**
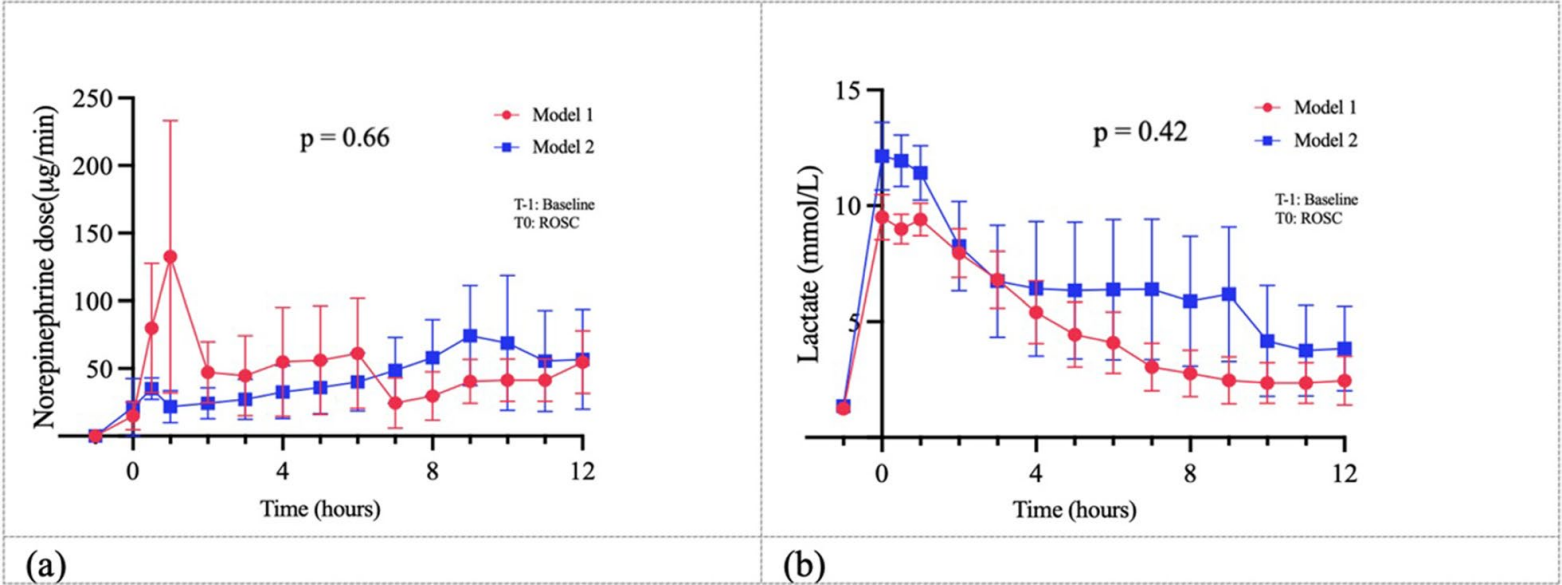
**a** Time-course of norepinephrine dose (μg/min) in the two models; **b** Time-course of blood lactate concentration (mmol/L) in the two models. Norepinephrine dose and blood lactate concentrations increased during the experiment without any significant difference between the two models; T0 = ROSC, T-1 = baseline (before the start of the CA procedure)

Six animals were needed to allow the testing of different interventions (e.g. installation of the animal, implementation of multimodal neuromonitoring, cannulas insertion, CA procedure and brain autopsy). Five swine were enrolled for each model (3 females and 2 males in each group). The average weight was 50 (3.1) kgs in the Model 1 and 52.4 (2.9) kgs in the Model 2. All 10 animals achieved ROSC and were therefore monitored in the post-resuscitation phase for 12 h (Table 1). Two animals died from refractory distributive shock before the end of the experiment (6 h after ROSC for one animal in the model 1 and 9 h after ROSC for one animal in the model 2). Both models were characterized by a severe distributive shock; norepinephrine doses and blood lactate concentrations were comparable between the two groups (Fig. 3).

**Table 1:** Comparison of main parameters between the two models during the experiment

	Time-points	Model 1	Model 2	P value
Heart rate (beat/min)	Baseline	92 (15)*	98 (10)	0.55
	T1	167 (38)	139 (16)	0.17
	T4	112 (20)	133 (19)	0.14
	T8	132 (30)	144 (27)	0.55
	T12	161 (48)	154 (36)	0.8
Mean arterial pressure (mmHg)	Baseline	78 (6)	69 (3)	0.02
	T1	61 (11)	69 (3)	0.16
	T4	68 (6)	71 (8)	0.65
	T8	68 (3)	68 (7)	0.97
	T12	65 (4)	64 (6)	0.77
PaO_2_ (mmHg)	Baseline	126 (3)	104 (22)	0.06
	T1	117 (28)	112 (16)	0.77
	T4	106 (16)	109 (6)	0.64
	T8	95 (33)	98 (14)	0.91
	T12	106 (5)	104 (13)	0.79
PaCO_2_ (mmHg)	Baseline	40 (3)	42 (3)	0.54
	T1	40 (10)	35 (2)	0.27
	T4	38 (10)	37 (4)	0.79
	T8	42 (8)	42 (2)	0.9
	T12	39 (1)	43 (4)	0.17

^*^Data are presented as mean (SD)

*Baseline* before the start of the CA procedure, *T1* 1 h after ROSC, *T4* 4 h after ROSC, *T8* 8 h after ROSC, *T12* 12 h after ROSC

**Fig. 4:**
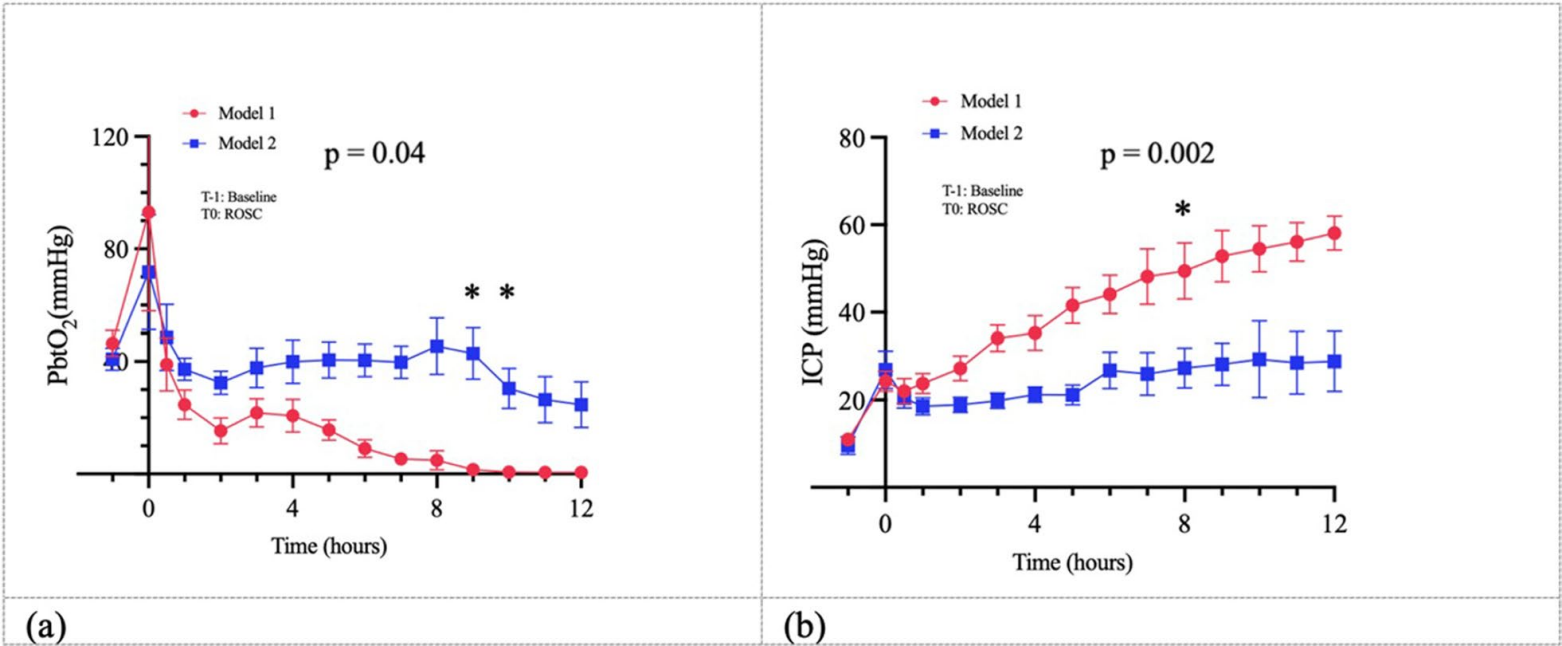
**a** Time-course of PbtO_2_ (mmHg) in the two models; **b** Time-course of ICP (mmHg) in the two models. PbtO2 values decreased and ICP levels increased during the experiment in both models, with these changes being significantly more pronounced in the first model; T0 = ROSC, T-1 = baseline (before the start of the CA procedure)

However, brain perfusion and oxygenation was more altered in the first model. Indeed, PbtO_2_ values decreased and ICP levels increased during the experiment in both models, but their alterations were significantly more pronounced in the first model (Fig. 4).

### Systematic review of the literature

**Fig. 5:**
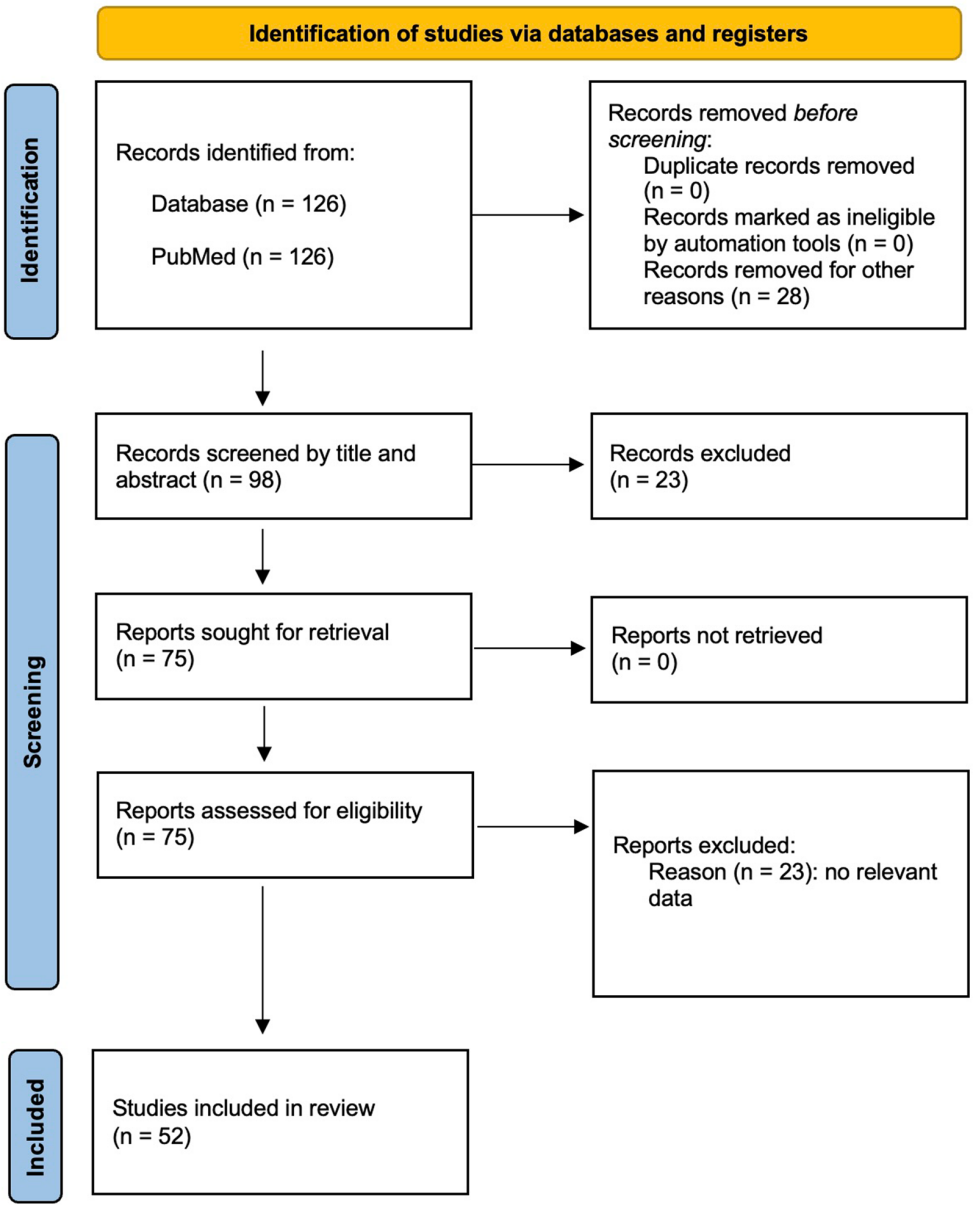
Flowchart of the systematic review. 126 records were identified from PubMed. 28 records were removed before screening. After exclusion of 23 records, 75 reports were assessed for eligibility. After exclusion of 23 reports because of no relevant data, 52 studies were included in the Systematic Review

The flowchart of the systematic review is reported in Fig. 5. Fifty-two studies published over the search period (from February 1, 2001, to September 1, 2022) were included (Supplementary material, table S2). With the exception of 4 studies performed in sheep and dogs [21–24], all other studies were performed in pigs.

VF induction with an electrical current (n = 40; 77%) or occlusion of coronary arteries (n = 7; 13%) were the most common methods to induce CA in these models. VF was mostly triggered by direct application of an electrical current on the endocardium or, less frequently, on the epicardium. For this purpose, a pacing wire was generally advanced, through an introducer inserted in the external jugular or in the femoral vein, until reaching the endocardium and then connected to an external battery. VF was confirmed by electrocardiogram and by the drop of the systemic arterial pressure.

Different strategies were used to induce coronary occlusion. After a left thoracotomy and pericardiotomy, the myocardial infarction was induced via the ligation of the mid left anterior descending coronary artery and VF was induced using an electrical current [21]. Another group used sternotomy and pericardiotomy, therefore making the ligation of the proximal left anterior descending coronary artery induced a myocardial infarction and due to proximal level of the coronary ligature, cardiogenic shock was systematically followed by a CA [25–28]. In another model, 15 min after a myocardial infarction induced by fluoroscopy-guided intravascular balloon occlusion of the proximal coronary circumflex artery, VF was induced using an electrical current [29]. Finally, 2 min after myocardial infarction induced by a hand-made pneumatic occluding device inflated around the proximal left descending coronary artery, VF was induced using an electrical current [30]. In few studies, asphyxia [31, 32], deep hypothermia [33, 34] and VF induction via injection of potassium [35] were used to induce CA.

The “CPR strategy”, which includes the no-flow time, the low-flow time, the chest compression technique, and the time to ECMO, also differed among studies. The no-flow time was extremely variable, ranging from 0 to 30 min. The low-flow time applied in selected studies ranged from 0 to 180 min. In 28 studies (54%), no CPR was performed. Manual or mechanical external chest compression were implemented in 16 studies (31%). Open chest massage was applied in 7 studies (13%). In one study [29], ECMO support was used to mimic flow generated by CPR.

With the exception of one study [36], the time from CA induction to the initiation of ECMO was consistently less than one hour. The duration of the entire experiment varied across protocols, ranging from few minutes to several days (more than 1 day, n = 9; 17%).

The outcomes studied in the fifty-one studies are displayed in the Supplementary material (Table S2 and S3). These outcomes cover a wide range of areas. General outcomes included the rate of ROSC (n = 12; 23%), survival rate (n = 9; 17%), extubation time, successful weaning from ECMO, and the feasibility of initiating ECMO during mechanical chest compression. Cardiac outcomes (n = 14; 27%) involved the study of electrophysiology, cardiac imaging using echography and magnetic resonance imaging (MRI), and histological evaluation of myocardial necrosis. Respiratory outcomes (n = 6; 11%) included the evaluation of lung vascular permeability and histological assessment of pulmonary inflammation. Hemodynamical outcomes (n = 21; 40%) encompassed the requirement for vasopressors, fluid resuscitation, microcirculation assessment, lactate clearance, and various hemodynamic measurements such as mean aortic pressure, left ventricle mean pressure, central venous pressure, pulmonary artery pressure, and pulmonary wedge pressure. Urinary outcomes (n = 15; 29%) included urine output, kidney function and histological evaluation of kidney function. Biomarkers outcomes (n = 28; 54%) consisted of troponin, myoglobin, creatine phosphokinase, alanine aminotransferase, aspartate aminotransferase, cystatin C, bilirubin, creatinine, blood nitrogen urea, and circulatory inflammatory mediators. Other outcomes were also considered including the evaluation of coagulation, immune function of the spleen, liver function, antibiotic kinetics, and assessment of ischemic enteric damage.

In the systematic review, 28 studies (54%) included the evaluation of neurological outcomes in their protocols (Supplementary material, Table S3). Various components of multimodal neuromonitoring are employed in these studies. Clinical evaluation (n = 8; 15%) involved the use of the Neurological Deficit Score (NDS) [37], which comprises five neurological examination components (central nerve function, respiration, motor sensory function, level of consciousness, and behavior). Each category can be assigned a maximum score of 100, with the general score representing the sum of all categories (ranging from 0 for normal to 500 for brain death). Brain perfusion was assessed through monitoring of PbtO_2_ (n = 2; 4%) and intracranial pressure (ICP) (n = 6; 11%) levels. Brain activity was evaluated using electroencephalography (EEG) (n = 4; 8%). Additional assessments (n = 23; 44%) include examination of brain biomarkers of cell damage (such as S100 β, UCHL1, GFAP, and NSE), near-infrared spectroscopy (NIRS) and bispectral index (BIS) monitoring, extracellular cerebral metabolites assessed by cerebral microdialysis, regional cerebral blood flow, carotid blood flow measurement, brain magnetic resonance imaging (MRI), evaluation of somatosensory-evoked potentials, transcranial Doppler ultrasound (TCD), and monitoring of jugular venous oxygen saturation (SjO_2_). Furthermore, brain tissues were harvested in some experimental models (n = 7; 13%). In addition to study brain morphology, assessments of neuronal damage, inflammation, and apoptosis were conducted using various biomolecular techniques such as enzyme-linked immunosorbent assay (ELISA), Western blotting, quantitative real-time polymerase chain reaction (qRT-PCR), and immunohistochemistry (IHC).

## Discussion

In this study, we first reported the results of two different animal models of experimental refractory CA implementing ECPR developed in our laboratory. Second, we performed a systematic review of all existing ECPR large animal models published in the literature.

Development of experimental models for ECPR is essential to evaluate potential positive and adverse effects of different therapies [1]. However, experimental models employed in ECPR studies exhibit heterogeneity, especially concerning the no-flow and low-flow times, making direct comparisons challenging. Depending on the outcomes to be evaluated, no-flow time duration may have varying impacts [2]. Notably, in the examination of neurological outcomes, no-flow time duration plays a significant role. Prolonged periods of no-flow correlate with worse survival rates and neurological prognoses, primarily due to the vulnerability of brain cells, especially neurons, during cardiac arrest (CA) [12–14]. Cessation of cardiac output during the no-flow phase leads to cerebral ischemia, exacerbating neuronal damage. In a swine model of CA, an 8-min period of no-flow resulted in a 30–40% mortality rate within 60 h of post-resuscitation care, while a 6-min period did not result in any deaths [38–40]. Similarly, the duration of low-flow time significantly influences neurological outcomes, as partial restoration of carotid blood flow may not adequately maintain neuronal integrity, leading to additional ischemic injury [14]. In our study, we observed that model 1, subjected to a longer no-flow time, exhibited more pronounced impairment in brain perfusion compared to model 2. Therefore, careful consideration of both no-flow and low-flow durations is crucial for effectively studying brain function in animal experiments.

Most studies included in our systematic review are conducted on pigs, which are considered an excellent experimental model for several reasons [41–43]: their size allows for the use of human monitoring devices and similar drug dosages; they tolerate surgical procedures well; their physiological parameters are similar to humans; they provide ample blood volume for multiple sample collections; their large brains facilitate brain tissue samplings; their wide chests allow for the use of mechanical chest compression devices and external defibrillators; and neurological scores have been developed in pigs to assess neurobehavioral functions.

The two most common methods used to induce CA are VF induction with an electrical current and occlusion of coronary arteries. VF induction offers advantages in terms of reproducibility and precise CA onset determination. However, occlusion of coronary arteries, mimicking the most common cause of CA in the population, has its drawbacks, including technical difficulty, variability in inducing CA, and delayed restoration of coronary blood flow, potentially exacerbating myocardial and systemic inflammation [12–14]. In most of the studies analyzed in our systematic review, CPR was not performed. When CPR was conducted, it involved external chest compressions or open chest massage, depending on the CA induction method. External chest compression offers several advantages over open chest massage, including less invasiveness and closer alignment with real-life resuscitation practices. In some cases, ECMO support was used to mimic the flow generated by CPR, although it deviates from standard practice [27].

According to the European Resuscitation Guidelines for advanced life support published in 2021 [44], ECPR may be considered as a rescue therapy for selected patients with cardiac arrest when conventional CPR is failing, provided it can be implemented [44]. However, in 28 studies (54%), no CPR was performed, complicating the translation of these models to clinical practice. Additionally, another critical criterion is the time required to establish ECPR, which should be less than 60 min from the initiation of CPR. In all studies except one, the time elapsed from CA induction to the initiation of ECMO was less than one hour [36]. In the model 2, we selected no-flow and low-flow (CPR) durations to closely mimic real-life situations.

A wide range of outcomes were assessed in the studies included in our review. Depending on the outcomes considered, the duration of the entire experiment varied, ranging from minutes to days. Longer protocols offer numerous advantages; besides measuring survival rates, they facilitate the evaluation of potential effects of therapies on brain and cardiac histology, as well as neurobehavioral functions. Moreover, longer protocols are more reflective of clinical practice. However, most studies (n = 43; 83%) included in our review had a protocol duration of less than 1 day. This may be attributed to the challenges associated with longer protocols, such as the need for additional staff, infrastructure, and budget. Furthermore, the risk of animal mortality before the experiment's completion is not insignificant.

## Conclusions

In this study, we described two different experimental models of ECPR developed in our laboratory and we provided a systematic review of all animal models described in the literature. This study underscores the crucial role played by the duration of no-flow and low-flow in influencing the severity of brain injuries following ECPR. Nonetheless, the diverse experimental models employed in ECPR studies present large heterogeneities, limit direct comparisons and external validation of reported findings. It remains imperative to standardize the experimental models of ECPR to promote consistency and improve comparability across studies.

## Supplementary Information

Additional file 1.

## Data Availability

The datasets used and/or analysed during the current study are available from the corresponding author on reasonable request.

## Abbreviations

CA: Cardiac arrest
CPR: Cardiopulmonary resuscitation
CT: Cerebral temperature
ECMO: Extracorporeal cardiopulmonary resuscitation
EEG: Electroencephalography
ECPR: Extracorporeal cardiopulmonary resuscitation
FiO_2_: Fraction of inspired oxygen
HIBI: Hypoxic-ischemic brain injury
ICP: Intracranial pressure
MRI: Magnetic resonance imaging
MAP: Mean arterial pressure
MNM: Multimodal neuromonitoring
NDS: Neurological deficit score
OHCA: Out-of-hospital cardiac arrest
PCAS: Post-cardiac arrest syndrome
PaCO_2_: Partial pressure of carbon dioxide
PaO_2_: Partial pressure of oxygen
PbtO_2_: Brain oxygen pressure
ROSC: Return of spontaneous circulation
VF: Ventricular fibrillation
