# Supplementary material for: Extracorporeal cardiopulmonary resuscitation: a comparison of two experimental approaches and systematic review of experimental models
Source: Intensive Care Med Exp. 2024 Sep 13;12:80. doi: 10.1186/s40635-024-00664-1 (PMC11399547; doi:10.1186/s40635-024-00664-1)
Supplement: Supplementary file 1 — Additional file 1. [file 40635_2024_664_MOESM1_ESM.docx]

Table S1: PubMed search strategy according to the Systemic Review Centre for Laboratory Animal Experimentation (SYRCLE) grading system ^1,2^

1. de Vries RB, Hooijmans CR, Tillema A, Leenaars M, Ritskes-Hoitinga M. Updated version of the Embase search filter for animal studies. Lab Anim. 2014 Jan;48(1):88.
2. Hooijmans CR, Tillema A, Leenaars M, Ritskes-Hoitinga M. Enhancing search efficiency by means of a search filter for finding all studies on animal experimentation in PubMed. *Lab Anim* 2010; 44: 170–175.

| **Search number** |  | **Results** |
| --- | --- | --- |
| Search N°1 (#1) | (CPR [Title/Abstract] OR Resuscitation [Title/Abstract] OR Cardiopulmonary Resuscitation [MeSH Terms]) AND (ECMO[Title/Abstract] OR extracorporeal membrane oxygenation[Title/Abstract] OR ECPR[Title/Abstract] OR E-CPR[Title/Abstract] OR extracorporeal cardiopulmonary resuscitation[Title/Abstract] OR ECLS[Title/Abstract] OR extracorporeal membrane oxygenation [MeSH Terms]) | 2982 |
| Search N°2 (#2) | ("animal experimentation"[MeSH Terms] OR "models, animal"[MeSH Terms] OR "invertebrates"[MeSH Terms] OR "Animals"[Mesh:noexp] OR "animal population groups"[MeSH Terms] OR "chordata"[MeSH Terms:noexp] OR "chordata, nonvertebrate"[MeSH Terms] OR "vertebrates"[MeSH Terms:noexp] OR "amphibians"[MeSH Terms] OR "birds"[MeSH Terms] OR "fishes"[MeSH Terms] OR "reptiles"[MeSH Terms] OR "mammals"[MeSH Terms:noexp] OR "primates"[MeSH Terms:noexp] OR "artiodactyla"[MeSH Terms] OR "carnivora"[MeSH Terms] OR "cetacea"[MeSH Terms] OR "chiroptera"[MeSH Terms] OR "elephants"[MeSH Terms] OR "hyraxes"[MeSH Terms] OR "insectivora"[MeSH Terms] OR "lagomorpha"[MeSH Terms] OR "marsupialia"[MeSH Terms] OR "monotremata"[MeSH Terms] OR "perissodactyla"[MeSH Terms] OR "rodentia"[MeSH Terms] OR "scandentia"[MeSH Terms] OR "sirenia"[MeSH Terms] OR "xenarthra"[MeSH Terms] OR "haplorhini"[MeSH Terms:noexp] OR "strepsirhini"[MeSH Terms] OR "platyrrhini"[MeSH Terms] OR "tarsii"[MeSH Terms] OR "catarrhini"[MeSH Terms:noexp] OR "cercopithecidae"[MeSH Terms] OR "hylobatidae"[MeSH Terms] OR "hominidae"[MeSH Terms:noexp] OR "gorilla gorilla"[MeSH Terms] OR "pan paniscus"[MeSH Terms] OR "pan troglodytes"[MeSH Terms] OR "pongo pygmaeus"[MeSH Terms]) OR ((animals[tiab] OR animal[tiab] OR mice[Tiab] OR mus[Tiab] OR mouse[Tiab] OR murine[Tiab] OR woodmouse[tiab] OR rats[Tiab] OR rat[Tiab] OR murinae[Tiab] OR muridae[Tiab] OR cottonrat[tiab] OR cottonrats[tiab] OR hamster[tiab] OR hamsters[tiab] OR cricetinae[tiab] OR rodentia[Tiab] OR rodent[Tiab] OR rodents[Tiab] OR pigs[Tiab] OR pig[Tiab] OR swine[tiab] OR swines[tiab] OR piglets[tiab] OR piglet[tiab] OR boar[tiab] OR boars[tiab] OR "sus scrofa"[tiab] OR ferrets[tiab] OR ferret[tiab] OR polecat[tiab] OR polecats[tiab] OR "mustela putorius"[tiab] OR "guinea pigs"[Tiab] OR "guinea pig"[Tiab] OR cavia[Tiab] OR callithrix[Tiab] OR marmoset[Tiab] OR marmosets[Tiab] OR cebuella[Tiab] OR hapale[Tiab] OR octodon[Tiab] OR chinchilla[Tiab] OR chinchillas[Tiab] OR gerbillinae[Tiab] OR gerbil[Tiab] OR gerbils[Tiab] OR jird[Tiab] OR jirds[Tiab] OR merione[Tiab] OR meriones[Tiab] OR rabbits[Tiab] OR rabbit[Tiab] OR hares[Tiab] OR hare[Tiab] OR diptera[Tiab] OR flies[Tiab] OR fly[Tiab] OR dipteral[Tiab] OR drosphila[Tiab] OR drosophilidae[Tiab] OR cats[Tiab] OR cat[Tiab] OR carus[Tiab] OR felis[Tiab] OR nematoda[Tiab] OR nematode[Tiab] OR nematoda[Tiab] OR nematode[Tiab] OR nematodes[Tiab] OR sipunculida[Tiab] OR dogs[Tiab] OR dog[Tiab] OR canine[Tiab] OR canines[Tiab] OR canis[Tiab] OR sheep[Tiab] OR sheeps[Tiab] OR mouflon[Tiab] OR mouflons[Tiab] OR ovis[Tiab] OR goats[Tiab] OR goat[Tiab] OR capra[Tiab] OR capras[Tiab] OR rupicapra[Tiab] OR chamois[Tiab] OR haplorhini[Tiab] OR monkey[Tiab] OR monkeys[Tiab] OR anthropoidea[Tiab] OR anthropoids[Tiab] OR saguinus[Tiab] OR tamarin[Tiab] OR tamarins[Tiab] OR leontopithecus[Tiab] OR hominidae[Tiab] OR ape[Tiab] OR apes[Tiab] OR pan[Tiab] OR paniscus[Tiab] OR "pan paniscus"[Tiab] OR bonobo[Tiab] OR bonobos[Tiab] OR troglodytes[Tiab] OR "pan troglodytes"[Tiab] OR gibbon[Tiab] OR gibbons[Tiab] OR siamang[Tiab] OR siamangs[Tiab] OR nomascus[Tiab] OR symphalangus[Tiab] OR chimpanzee[Tiab] OR chimpanzees[Tiab] OR prosimians[Tiab] OR "bush baby"[Tiab] OR prosimian[Tiab] OR bush babies[Tiab] OR galagos[Tiab] OR galago[Tiab] OR pongidae[Tiab] OR gorilla[Tiab] OR gorillas[Tiab] OR pongo[Tiab] OR pygmaeus[Tiab] OR "pongo pygmaeus"[Tiab] OR orangutans[Tiab] OR pygmaeus[Tiab] OR lemur[Tiab] OR lemurs[Tiab] OR lemuridae[Tiab] OR horse[Tiab] OR horses[Tiab] OR pongo[Tiab] OR equus[Tiab] OR cow[Tiab] OR calf[Tiab] OR bull[Tiab] OR chicken[Tiab] OR chickens[Tiab] OR gallus[Tiab] OR quail[Tiab] OR bird[Tiab] OR birds[Tiab] OR quails[Tiab] OR poultry[Tiab] OR poultries[Tiab] OR fowl[Tiab] OR fowls[Tiab] OR reptile[Tiab] OR reptilia[Tiab] OR reptiles[Tiab] OR snakes[Tiab] OR snake[Tiab] OR lizard[Tiab] OR lizards[Tiab] OR alligator[Tiab] OR alligators[Tiab] OR crocodile[Tiab] OR crocodiles[Tiab] OR turtle[Tiab] OR turtles[Tiab] OR amphibian[Tiab] OR amphibians[Tiab] OR amphibia[Tiab] OR frog[Tiab] OR frogs[Tiab] OR bombina[Tiab] OR salientia[Tiab] OR toad[Tiab] OR toads[Tiab] OR "epidalea calamita"[Tiab] OR salamander[Tiab] OR salamanders[Tiab] OR eel[Tiab] OR eels[Tiab] OR fish[Tiab] OR fishes[Tiab] OR pisces[Tiab] OR catfish[Tiab] OR catfishes[Tiab] OR siluriformes[Tiab] OR arius[Tiab] OR heteropneustes[Tiab] OR sheatfish[Tiab] OR perch[Tiab] OR perches[Tiab] OR percidae[Tiab] OR perca[Tiab] OR trout[Tiab] OR trouts[Tiab] OR char[Tiab] OR chars[Tiab] OR salvelinus[Tiab] OR "fathead minnow"[Tiab] OR minnow[Tiab] OR cyprinidae[Tiab] OR carps[Tiab] OR carp[Tiab] OR zebrafish[Tiab] OR zebrafishes[Tiab] OR goldfish[Tiab] OR goldfishes[Tiab] OR guppy[Tiab] OR guppies[Tiab] OR chub[Tiab] OR chubs[Tiab] OR tinca[Tiab] OR barbels[Tiab] OR barbus[Tiab] OR pimephales[Tiab] OR promelas[Tiab] OR "poecilia reticulata"[Tiab] OR mullet[Tiab] OR mullets[Tiab] OR seahorse[Tiab] OR seahorses[Tiab] OR mugil curema[Tiab] OR atlantic cod[Tiab] OR shark[Tiab] OR sharks[Tiab] OR catshark[Tiab] OR anguilla[Tiab] OR salmonid[Tiab] OR salmonids[Tiab] OR whitefish[Tiab] OR whitefishes[Tiab] OR salmon[Tiab] OR salmons[Tiab] OR sole[Tiab] OR solea[Tiab] OR "sea lamprey"[Tiab] OR lamprey[Tiab] OR lampreys[Tiab] OR pumpkinseed[Tiab] OR sunfish[Tiab] OR sunfishes[Tiab] OR tilapia[Tiab] OR tilapias[Tiab] OR turbot[Tiab] OR turbots[Tiab] OR flatfish[Tiab] OR flatfishes[Tiab] OR sciuridae[Tiab] OR squirrel[Tiab] OR squirrels[Tiab] OR chipmunk[Tiab] OR chipmunks[Tiab] OR suslik[Tiab] OR susliks[Tiab] OR vole[Tiab] OR voles[Tiab] OR lemming[Tiab] OR lemmings[Tiab] OR muskrat[Tiab] OR muskrats[Tiab] OR lemmus[Tiab] OR otter[Tiab] OR otters[Tiab] OR marten[Tiab] OR martens[Tiab] OR martes[Tiab] OR weasel[Tiab] OR badger[Tiab] OR badgers[Tiab] OR ermine[Tiab] OR mink[Tiab] OR minks[Tiab] OR sable[Tiab] OR sables[Tiab] OR gulo[Tiab] OR gulos[Tiab] OR wolverine[Tiab] OR wolverines[Tiab] OR minks[Tiab] OR mustela[Tiab] OR llama[Tiab] OR llamas[Tiab] OR alpaca[Tiab] OR alpacas[Tiab] OR camelid[Tiab] OR camelids[Tiab] OR guanaco[Tiab] OR guanacos[Tiab] OR chiroptera[Tiab] OR chiropteras[Tiab] OR bat[Tiab] OR bats[Tiab] OR fox[Tiab] OR foxes[Tiab] OR iguana[Tiab] OR iguanas[Tiab] OR xenopus laevis[Tiab] OR parakeet[Tiab] OR parakeets[Tiab] OR parrot[Tiab] OR parrots[Tiab] OR donkey[Tiab] OR donkeys[Tiab] OR mule[Tiab] OR mules[Tiab] OR zebra[Tiab] OR zebras[Tiab] OR shrew[Tiab] OR shrews[Tiab] OR bison[Tiab] OR bisons[Tiab] OR buffalo[Tiab] OR buffaloes[Tiab] OR deer[Tiab] OR deers[Tiab] OR bear[Tiab] OR bears[Tiab] OR panda[Tiab] OR pandas[Tiab] OR "wild hog"[Tiab] OR "wild boar"[Tiab] OR fitchew[Tiab] OR fitch[Tiab] OR beaver[Tiab] OR beavers[Tiab] OR jerboa[Tiab] OR jerboas[Tiab] OR capybara[Tiab] OR capybaras[Tiab]) NOT medline[subset]) | 8,069,792 |
| Search N°3 (#1 AND #2) |  | 126 |
| Search N°4 (#1 AND #2 between 2001 and 2022) |  | 98 |
|  |  |  |

Table S2: experimental protocols using ECPR in refractory cardiac arrest

| **Study group** | **Species** | **N** | **Type of CA** | **CPR strategy** | **Model duration** | **Main outcomes** |
| --- | --- | --- | --- | --- | --- | --- |
| *Stub*  *et al.* [19] | Sheep | 12 | Ligation of the mid LAD artery  VF induction by electric shock | No flow: 3 min  Low flow: 10 min  CC: open chest  Time to ECMO: 13 min | Until ROSC | CPP  ROSC  VF amplitude |
| *Ao*  *et al.* [20] | Dog | 17 | VF induction by electric shock | No flow: 15 min  Low flow: 0 min  Time to ECMO: 15 min | ECMO period (24h)  +  ICU stay (72h) | Evaluation of degenerating pyramidal hippocampal neurons at brain biopsy  Evaluation of necrotic myocardial mass  Extubation time  Mortality  NDS  Requirements for vasopressors  Time to ROSC |
| *Ichinose*  *et al.* [21] | Dog | 12 | VF induction by electric shock | No flow: 15 min  Low flow: 0 min  Time to ECMO: 15 min | ECMO period (24h)  +  ICU stay (96h) | Administration doses of catecholamines and fluid during resuscitation  Changes in the ACT at 1, 3, 6, 12 and 24h after resuscitation  Hematocrit and platelet count during resuscitation at 1, 6, 12 and 24h after resuscitation  NDS  Postmortal macroscopic examination  Survival rate after 120h of resuscitation |
| *Li*  *et al.* [22] | Dog | 10 | VF induction by electric shock | No flow: variable  Low flow: 0 min  Time to ECMO: 31.2 ± 9.7 min in the nonpulsatile group vs 28.7 ± 11.4 min in the pulsatile group | ECMO period (6h) | Endothelial integrity  Microcirculatory perfusion  Pulmonary inflammation |
| *Prague*  *Group* [45-51] | Pig | 23 | VF induction by electric shock | No flow: 20 min  Low flow: 0 min  Time to ECMO: 20 min | ECMO period (90 min) | Cerebral oxygenation (NIRS)  Levels of NSE and Cystatin C  Levels of troponin I, myoglobin, CPK, and ALT  Levels of reactive oxygen metabolites |
|  | Pig | 18 | VF induction by electric shock | No flow: 20 min  Low flow: 0 min  Time to ECMO: 20 min | ECMO period (60 min) | Brain (EEG and BIspectral Index) and cardiac electrical activity assessment  Peripherical tissue oxygen saturation evaluation  ROSC rate |
|  | Pig | 6 | VF induction by electric shock | No flow: 0 min  Low flow: 0 min  Time to ECMO: 0 min | ECMO period (180 min) | Study of Inducibility of VF, effective refractory period of the ventricles, QTc interval and potassium plasma levels |
|  | Pig | 26 | VF induction by electric shock | No flow: 15 min  Low flow: 0 min  Time to ECMO: 15 min  Defibrillation attempts | ECMO period (60 min)  +  Period between defibrillation and ROSC or death (variable) | Carotid and coronary blood flows assessment (Doppler flow wire)  Cerebral and peripherical oxygenation evaluation (NIRS)  Determination of median frequency of VF from right ventricular apex, CPP, myocardial oxygen metabolism and resuscitability  ECMO circuit, aortic, pulmonary arterial, and tracheal aspirate concentrations of antibiotics measurements (vancomycin, gentamicin and amikacin) at 30 and 60 min after administration + calculation of penetration ratios |
| *Oslo – Bergan group* [52, 53] | Pig | 28 | VF induction by electric shock | No flow: 15 min  Low flow: 0 min  Time to ECMO: 15 min | ECMO period (150 min)  +  ECMO weaning (60 min)  +  Stabilization period (60 min) | Comparison of cardiac MRI and hemodynamic measurements (pre- vs post arrest)  Myocardial injury estimation by serum concentrations of TroponinT and ASAT  Resuscitation success rate |
| *Nancy group*  [23-26] | Pig | 14 | Proximal ligation of the LAD artery | No flow: 0 min  Low flow: 20 min  CC: open chest  Time to ECMO: 20 min | ECMO period (6h) | Evaluation of renal and liver function  Lactate level at T0 and T6  Sublingual microcirculation evaluation  Total infused fluid |
|  | Pig | 18 | Proximal ligation of the LAD artery | No flow: 90 sec  Low flow: 40 min  CC: open chest  Time to ECMO: 41 min + 30 sec | ECMO period (6h) | Biological markers (creatinine, blood nitrogen urea, troponin, AST, ALT) and circulatory inflammatory mediators (IL-1β, IL-4, IL-6, IL-8, IL-10, IL- 12, GM-CSF, IFNδ, TGFβ and TNF-α) assessment  Hemodynamic parameters evaluation (carotid blood flow, amount of infused fluid, amount of administered norepinephrine, vascular reactivity, urine output)  Lactate clearance  Microcirculatory parameters (variations in sublingual microcirculation, regional tissue oxygenation) |
|  | Pig | 38 | Proximal ligation of the LAD artery | No flow: 90 sec  Low flow: 30 min  CC: open chest  Time to ECMO: 31 min + 30 sec | ECMO period (6h) | Carotid blood flow assessment  Catecholamine requirement  Ischemic enteric damage evaluation  Laboratory measurements (creatinine, AST, ALT, bilirubine, troponin)  Lactate clearance  Lung vascular permeability (wet/dry weight ratio) evaluation  Regional tissue oxygenation evaluation (NIRS)  Sublingual microcirculation evaluation  Total infused fluid |
| *Beijing group* [54-58] | Pig | 44 | VF induction by electric shock | No flow: 8 min  Low flow: 6min  CC: conventional CPR  Time to ECMO: 14 min | ECMO period (6h) | Evaluation of myocardial injury and myocardial apoptosis (blood analysis, echography, microscopy, immunohistochemistry, qRT-PCR and immunofluorescence staining assay)  Immune function of spleen  ROSC  Reactive oxygen species levels  Successful weaning of ECMO  6h survival rate after ROSC |
|  | Pig | 16 | VF induction by electric shock | No flow: 12 min  Low flow: 2 min  CC: conventional CPR  Time to ECMO: 14 min | 6h post ROSC | Evaluation of extravascular lung water and pulmonary vascular permeability index  Evaluation of lung morphology using electron microscopy  Survival rate  Study of blood and tissue biomarkers (lung) |
|  | Pig | 16 | VF induction by electric shock | No flow: 12 min  Low flow: 2 min (CPR alone) + 4 min (CPR combined with ECMO)  CC: conventional CPR  Time to ECMO: 14 min | 6h post ROSC | Evaluation of kidney function (serum creatinine, urine output, serum and urine acute kidney injury biomarkers, renal histopathology examination, renal apoptosis assessment)  Survival rate |
|  | Pig | 16 | VF induction by electric shock | No flow: 0 min  Low flow: 10 min  CC: conventional CPR  Time to ECMO: 10 min | 6h post ROSC | Brain histology  Evaluation of inflammation and ATPase activity in pig brain  Survival rate |
| *Freiburg – Foerster group* [59-63] | Pig | 37 | VF induction by electric shock | No flow: 20 min  Low flow: 0 min  Time to ECMO: 20 min | ECMO period (1h)  +  ICU stay (7 days / 8 days) | Daily NDS  Dosage of AST, ALT, CK, CK-MB, NSE, Bilirubin, urea, creatinine during the protocol  Lactate clearance  Survival rate |
|  | Pig | 24 | VF induction by electric shock | No flow: 15 min  Low flow: 0 min  Time to ECMO: 15 min | ECMO period (60 min)  +  ICU stay (7 days) | Brain histological examination  Brain MRI evaluation  Daily NDS  Dosage of AST, ALT, CK-MB, NSE during the protocol  EEG  Hemodynamic parameters assessment (cardiac output, systemic and pulmonary vascular resistance indices) |
|  | Pig | 35 | VF induction by electric shock | No flow: variable (0 vs 15 vs 20 min)  Low flow: variable (0 vs 10 vs 15 min)  CC: open chest (2)  Time to ECMO: variable (15 vs 20 vs 25 min) | ECMO period (60 min)  +  ICU stay (7 days) | Brain injury estimated with MRI  Daily NDS  Markers of cellular injury assessment (ALT, AST, NSE) |
| *Freiburg - Wollborn*  *Group* [29, 30] | Pig | 29 | Asphyxia | No flow: 4.5 min  Low flow: 5 min  CC: conventional CPR  Time to ECMO: 9.5 min | ECMO period (5h and 50min) | Cardiac performance analysis using echography and thermodilution techniques  Cerebral function evaluation using MNM (somatosensory-evoked potentials, NIRS, TCD)  Histological and serological myocardial damage markers  Histopathologic brain damage and molecular markers  Microvascular function |
| *Karlsen*  *et al.* [27] | Pig | 20 | Myocardial infarction induced by occlusion of the proximal coronary circumflex artery  VF induction by electric shock (after 15 min of occlusion) | No flow: 10 min  Low flow: ECMO support for 5-10 min (to mimic flow generated by CPR)  Defibrillation after 5min of ECMO | High-flow ECMO support (60 min)  +  ECMO weaning (30 min)  +  Stabilization period (30 min) | Cardiac function pre- and post-arrest assessed by MRI and invasive pressure measurements  Myocardial injury estimated with MRI, triphenyl tetrazolium chloride staining and serum concentrations of cardiac troponin T |
| *Zhang*  *et al.* [36] | Pig | 18 | VF induction by electric shock | No flow: 6 min  Low flow: 0 min  Time to ECMO: 6 min | ECMO period (32h) | Apoptosis investigation in the cerebral frontal cortex |
| *Paris*  *Group*  [28, 64, 65] | Pig | 24 | VF induction by electric shock | No flow: 15 min  Low flow: 0 min  Time to ECMO: 15 min | ECMO period (30 min)  +  ICU stay (120 min) | Blood levels of ALT, creatinine, troponin, PS100, IL-1α and IL-1β  CBF  ICP  NIRS  PRx |
|  | Pig | 18 | Coronary artery occlusion  VF induction by electric shock | No flow: 5 min  Low flow: 15 min  CC: conventional CPR  Time to ECMO: 20 min | ECMO period (240 min) | Blood levels of lactate, creatinine, troponine I, protein S100, and ALAT  Carotid blood flow  Infarct size  Rate of ROSC and of return of spontaneous heartbeat |
| *Mehaffey*  *et al.* [66] | Pig | 15 | VF induction by electric shock | No flow: 20 min  Low flow: 0 min  Time to ECMO: 20 min | ECMO period (6h) | Ability to wean off ECMO  Evaluation of renal (urine concentration of Neutrophil gelatinase-associated lipocalin), hepatic (plasma concentration of AST) and cardiac (plasma concentration of Troponin I) injury  Fluid requirements  Severity of systemic reperfusion injury (lactate clearance, plasma concentration of TNF- α, IFN-δ, IL-4 and IL-10) |
| *Grenoble group* [31, 32] | Pig | 44 | CA induced with deep hypothermia | No flow: 30 min  Low flow: 0 min  Time to ECMO: 30 min | ECMO period (till body temperature reached 35°C) | Assessement of the cardiac and pulmonary pathophysiological response during the cooling phase and the rewarming phase  Blood levels of pro- inflammatory cytokines (IL-1β, IL-6, IL-10 and TNF-α) and of the receptor for advanced glycation end products  Evaluation of lung injury (capillary-alveolar permeability, distal alveolar fluid clearance, wet-to-dry weight ratio)  NSE dosage^32^ |
| *Oslo - Packer group* [67, 68] | Pig | 20 | VF induction by electric shock | No flow: 0 min  Low flow: 0 min  Time to ECMO: 0 min | ECMO period (40 min)  +  If ROSC after defibrillation attempts, ECMO removal and monitoring for 60 min | Cardiac autopsy  Cardiac function assessed by echography  Hemodynamic measurements (mean aortic pressure, CPP, carotid flow, left ventricle mean pressure, central venous pressure)  ROSC rate  Tissue perfusion evaluation (brain, kidney, ventricle, ileum) with fluorescent microsphere injections  Troponin-T and lactate levels assessement |
|  | Pig | 24 | VF induction by electric shock | No flow: 0 min  Low flow: 0 min  Time to ECMO: 0 min | ECMO period (60 min)  +  Weaning period (10 min)  +  Observation period (170 min) | Cardiac function assessed by echography  Hemodynamic measurements (device output, mean aortic- and left ventricular pressure, CPP)  ROSC rate after maximum 3 defibrillations  Tissue perfusion evaluation (brain, kidney, ventricle) with fluorescent microsphere injections and mean left descending artery + pulmonary flow  Troponin-T and lactate levels assessment |
| *Spinelli*  *et al.* [69] | Pig | 15 | VF induction by electric shock | No flow: 30 min  Low flow: 0 min  Time to ECMO: 30 min | ECMO period (6h) | Cardiovascular recovery (success of defibrillation, return of spontaneous heart beat, weanability from ECMO, left ventricular systolic function after weaning)  Cerebral recovery using MNM and autopsy  Lactate clearance |
| *Lee*  *et al.* [70] | Pig | 6 | VF induction by electric shock | No flow: 10 min  Low flow: 0 min  Time to ECMO: 10 min | ECMO period (3min) | Evaluation of the frequency component changes in the electrocardiogram by ECPR during prolonged VF |
| *Nilsen*  *et al.* [34] | Pig | 8 | VF induction by electric shock | No flow: 0 min  Low flow: 180 min  CC: conventional CPR  Time to ECMO: 180 min | ECMO period (till ROSC) | Global + cerebral O_2_ transport/extraction  Hemodynamic measurements assessment (cardiac output, mean arterial pressure)  Organ blood flow measurements using microspheres  Serum biomarkers levels for organ function and organ injury (brain, heart, kidney, liver, pancreas) |
| *Oslander*  *et al.* [71] | Pig | 10 | VF induction by electric shock | No flow: 0 min  Low flow: 45 min  CC: conventional CPR  Time to ECMO: 45 min | ECMO period (180 min) | Brain function (ICP, plasma level of S100 β, histology)  Hemodynamic parameters (cardiac output, CBF, MAP, oxygen delivery/uptake)  Kidney function (urinary output, plasma concentration of Neutrophil gelatinase-associated lipocalin, histology)  Metabolic parameters (PH, standard base excess, lactate)  Plasma concentration of IL-6 and TNF α |
| *Reynolds*  *et al.* [72] | Pig | 8 | VF induction by electric shock | No flow: variable (8 vs 15 min)  Low flow: variable (30, 40, 50 or 60 min)  CC: conventional CPR  Time to ECMO: variable | ECMO period (240 min)  +  Weaning ECMO period (60 min) | Hemodynamic parameters evaluation (MAP, CPP, VF waveform measures)  ROSC rate  Successful ECMO weaning  1 hour survival after weaning |
| *Mandigers*  *et al.* [73] | Pig | 6 | VF induction by electric shock | No flow: 5 min  Low flow: 30 min (8 min of basic life support, 22 min of advanced life support)  CC: conventional CPR  Time to ECMO: 35 min | ECMO period (120 min)  ICU stay (7 days) | Daily NDS  Skin mitochondrial partial oxygen pressure assessment |
| *Menegazzi*  *et al.* [74] | Pig | 5 | VF induction by electric shock | No flow: 8 min  Low flow: variable (till ECMO start)  CC: conventional CPR  Time to ECMO: Between 25 and 38 min | ECMO period (till EKG indicated shockable rhythm – 22, 32, 35, 44 and 65 min) | Feasibility of initiating extracorporeal life support during mechanical chest compression |
| *Weiser*  *et al.* [75] | Pig | 8 | VF induction by electric shock | No flow: 15 min  4°C aortic flush (150ml/kg): variable (mean time: 12 min)  Time to ECMO: variable (+/- 27 min) | Aortic flush period (variable)  +  ECMO period (max 60 min) | Feasibility of cooling the brain within a few minutes in a large pig cardiac arrest model |
| *Cremers*  *et al.* [33] | Pig | 8 | VF induction by injection of potassium | No flow: 2 min  Low flow: 0 min  Time to ECMO: 2 min | ECMO period (28 min) | Hemodynamic measurements assessment (pulmonary artery pressure, pulmonary wedge pressure, central venous pressure, mean arterial pressure, mean pulse pressure, mean coronary artery flow) |
| *Simonsen*  *et al.* [76] | Pig | 1 | VF induction by electric shock | No flow: 0 min  Low flow: 8 min  CC: conventional CPR  Time to ECMO: 8 min | 2h 17 min | Feasibility to cannulate and initiate veno-arterial ECMO treatment in airborne for cardiac arrest and severe CO intoxication |
| *Voicu*  *et al.* [77] | Pig | 13 | VF induction by electric shock | No flow: 10 min  Low flow: 20 min  CC: conventional CPR  Time to ECMO: 30 min | ECMO period (120 min)  +  Observation period (30 min) | Cardiac function assessment (echography, angiography, coronary pression pressure)  Hemodynamic parameters evaluation (arterial pressure, heart rate, central venous pressure, pulmonary capillary pressure) |
| *Bernhard*  *et al.* [78] | Pig | 7 | VF induction by electric shock | No flow: 5 min  Low flow: 30 min  CC: conventional CPR  Time to ECMO: 35 min | ECMO period (120 min) | Serum proteome profiles assessment |
| *Putzer*  *et al.* [79] | Pig | 14 | VF induction by electric shock | No flow: 8 min  Low flow: 0 min  Time to ECMO: 8 min | ECMO period (30 min) | Neurological assessment (ICP, laser-Doppler-derived regional cerebral blood flow, NIRS, PbtO2 and extracellular cerebral metabolites assessed by cerebral microdialysis) |

ACT = activated clotting time; ALT = alanine aminotransferase; AST = asparate aminotransferase; ATP = adenosine triphosphate ; CA = cardiac arrest; CBF = carotid blood flow; CC = chest compression; CPK = creatine-phosphokinase; CPP = coronary perfusion pressure; CPR = cardiopulmonary resuscitation; NDS = Neurologic Deficit Score (NDS 0% = normal, NDS 100% = brain death); ECMO = extracorporeal membrane oxygenation; ECPR = Extracorporeal CardioPulmonary Resuscitation; EEG = electroencephalography; EKG = electrocardiogram; GM-CSF = granulocyte-macrophage colony-stimulating factor; ICP = intracranial pressure; ICU = intensive care unit; IFN = interferon; IL = interleukin; LAD artery = left anterior descending artery; MAP = mean arterial pressure; MNM = multimodal neuromonitoring; MRI = magnetic resonance imaging; N = number; NIRS = near infrared spectroscopy; NSE = neuron-specific enolase; PbtO2 = brain tissue oxygen tension; PRx = pressure reactivity index; qRT-PCR = quantitative real-time polymerase chain reaction; ROSC = return of spontaneous circulation; TCD = transcranial doppler; TGF = transforming growth factor; TNF = tumor necrosis factor; VF = ventricular fibrillation

Table S3: neurological outcomes assessed in ECPR – cardiac arrest models

| **Study group** | **Clinical evaluation** | **ICP** | **Pbt02** | **CMD** | **EEG** | **Pathology** | **Others** |
| --- | --- | --- | --- | --- | --- | --- | --- |
| *Ao*  *et al.* [20] | NDS |  |  |  |  | Evaluation of degenerating pyramidal hippocampal neurons at brain biopsy | - |
| *Ichinose*  *et al.* [21] | NDS | - | - | - | - | - | - |
| *Prague*  *Group* [45, 47, 48, 50] | - | - | - | - | Brain electrical activity assessment | - | Brain (BIspectral Index) electrical activity assessment  CBF  NIRS  NSE dosage |
| *Nancy*  *Group* [23-25] | - | - | - | - | - | - | CBF |
| *Zhang*  *et al.* [54] | - | - | - | - | - | Brain histology and ultramicrostructure examination  Evaluation of inflammation (detection of IL-1, IL-1 β, IL-6, IL-10, TNF α, TGF β and KL-6 with ELISA and Western Blot) and ATPase activity (with colorimetric assays) in pig brain | - |
| *Freiburg – Foerster group* [59-63] | NDS | - | - | - | Evaluation of brain electrical activity | Evaluation of neuronal damage (frontal lobe, thalamus, striatum, hippocampus, brain stem and cerebellar hemispheres) | Brain MRI  NSE dosage |
| *Freiburg - Wollborn*  *group* [29] | - | - | - | - | - | Histopathologic brain damage and molecular markers (caspase-3 activity and heme oxygenase-1 expression) | NIRS  Somatosensory-evoked potentials  TCD |
| *Zhang*  *et al.* [36] | - | - | - | - | - | Apoptosis investigation (qRT-PCR, IHC, cellular morphology) in the cerebral frontal cortex | - |
| *Paris*  *Group*  [28, 64, 65] | - | yes | - | - | - | - | CBF  NIRS |
| *Grenoble*  *Group* [32] | - | - | - | - | - | - | NSE dosage |
| *Oslo - Packer group* [67, 68] | - | - | - | - | - | - | Brain blood flow rate  CBF |
| *Spinelli*  *et al.* [69] | - | Modification after CA and during ECPR | Modification after CA and during ECPR | - | Return of brain electrical activity | Quantification of brain hemorrhages and acute ischemic neurodegeneration | - |
| *Nielsen*  *et al.* [34] | - | yes | - | - | - | - | Cerebral oxygen delivery / uptake  Plasma biomarkers (S100 β, UCHL1, GFAP, NSE)  SjO2 |
| *Olander*  *et al.* [71] | - | yes | - | - | - | Evaluation of ischemic damage | Plasma level of S100 β |
| *Mandigers*  *et al.* [73] | NDS | - | - | - | - | - | - |
| *Putzer et al.* [79] | - | yes | yes | yes | - | - | Laser-Doppler-derived regional cerebral blood flow  NIRS |

ATP = adenosine triphosphate; BIS = BIspectral Index; CA = cardiac arrest; CBF = carotid blood flow; CMD = cerebral microdialysis; ECPR = extracorporeal cardiopulmonary resuscitation; EEG = electroencephalography; ELISA = Enzyme-linked immunosorbent assay; GFAP = glial fibrillary acidic protein; ICP = intracranial pressure; IHC = immunohistochemistry; IL = interleukin; MRI = magnetic resonance imaging; NDS = Neurologic Deficit Score (NDS 0% = normal, NDS 100% = brain death); NIRS = near infrared spectroscopy; NSE = neuron-specific enolase; qRT-PCR = quantitative real-time polymerase chain reaction; PbtO2 = brain tissue oxygen tension; TCD = transcranial doppler; TGF = transforming growth factor; TNF = tumor necrosis factor; UCHL1 = Ubiquitin carboxyl-terminal esterase L1; SjO2 = jugular venous oxygen saturation
